# Overexpression of proto-oncogene FBI-1 activates membrane type 1-matrix metalloproteinase in association with adverse outcome in ovarian cancers

**DOI:** 10.1186/1476-4598-9-318

**Published:** 2010-12-21

**Authors:** LiLi Jiang, Michelle KY Siu, Oscar GW Wong, Kar Fai Tam, Eric W-F Lam, Hextan YS Ngan, Xiao-Feng Le, Esther SY Wong, Hoi Yan Chan, Annie NY Cheung

**Affiliations:** 1 Department of Pathology and, The University of Hong Kong, HKSAR, China; 2 Department of Obstetrics and Gynaecology, The University of Hong Kong, HKSAR, China; 3 Department of Pathology, West China Hospital, Sichuan University, Chengdu, China; 4 Cancer Research UK laboratories, Department of Surgery and Cancer, Imperial College London, London, UK; 5 Department of Experimental Therapeutics, Division of Cancer Medicine, the University of Texas M. D. Anderson Cancer Center, Houston, Texas, USA

## Abstract

**Background:**

FBI-1 (factor that binds to the inducer of short transcripts of human immunodeficiency virus-1) is a member of the POK (POZ and Kruppel) family of transcription factors and play important roles in cellular differentiation and oncogenesis. Recent evidence suggests that FBI-1 is expressed at high levels in a subset of human lymphomas and some epithelial solid tumors. However, the function of FBI-1 in human ovarian cancers remains elusive.

**Results:**

In this study, we investigated the role of FBI-1 in human ovarian cancers, in particularly, its function in cancer cell invasion via modulating membrane type 1-matrix metalloproteinase (MT1-MMP). Significantly higher FBI-1 protein and mRNA expression levels were demonstrated in ovarian cancers samples and cell lines compared with borderline tumors and benign cystadenomas. Increased FBI-1 mRNA expression was correlated significantly with gene amplification (P = 0.037). Moreover, higher FBI-1 expression was found in metastatic foci (P = 0.036) and malignant ascites (P = 0.021), and was significantly associated with advanced stage (P = 0.012), shorter overall survival (P = 0.032) and disease-free survival (P = 0.016). *In vitro*, overexpressed FBI-1 significantly enhanced cell migration and invasion both in OVCA 420 and SKOV-3 ovarian carcinoma cells, irrespective of *p53 *status, accompanied with elevated expression of MT1-MMP, but not MMP-2 or TIMP-2. Moreover, knockdown of MT1-MMP abolished FBI-1-mediated cell migration and invasion. Conversely, stable knockdown of FBI-1 remarkably reduced the motility of these cells with decreased expression of MT1-MMP. Promoter assay and chromatin immunoprecipitation study indicated that FBI-1 could directly interact with the promoter spanning ~600bp of the 5'-flanking sequence of MT1-MMP and enhanced its expression in a dose-dependent manner. Furthermore, stable knockdown and ectopic expression of FBI-1 decreased and increased cell proliferation respectively in OVCA 420, but not in the p53 null SKOV-3 cells.

**Conclusions:**

Our results suggested an important role of FBI-1 in ovarian cancer cell proliferation, cell mobility, and invasiveness, and that FBI-1 can be a potential target of chemotherapy.

## Background

Ovarian cancer is the leading cause of death among gynecologic malignancies worldwide, and the survival rates remain disappointing for patients suffering from advanced cancers [1,2]. Development of cancer metastasis is the major cause that kills patients with ovarian cancer. However, the molecular mechanisms contributing to its aggressiveness are still not fully understood.

In the past decade, it has been established that the matrix metalloproteinases (MMPs), including Membrane-type-1 MMP (MT1-MMP or MMP14) and MMP-2, play a critical role in degrading the basement membrane and the extracellular matrix (ECM), resulting in tumor cell dissemination and outgrowth of secondary cancers [3,4]. MT1-MMP not only directly cleaves ECM components, but also functions as the main activator of MMP-2 [5,6]. Conversely, tissue inhibitor of metalloproteinase-2 (TIMP-2) inhibits MMP-2 after binding with its hemopexin domain [7,8]. As in other solid cancers, MT1-MMP has been reported to be widely expressed in ovarian cancers and related malignant ascites of all histological types, but not in normal ovarian epithelium or benign tumors [9-12]. Despite the central role of MT1-MMP in these cancer metastases, little is known about its transcriptional regulators [13].

FBI-1 (also known as POKEMON, LRF in mouse, or OCZF in rat), which was originally identified as a factor that binds to the inducer of short transcripts (IST) element of human HIV-1 genome [14], is a member of the POK (POZ and krǔppel) family of transcription factor. Recent reports revealed the participation of FBI-1 in NF-κB activation [15], adipogenesis [16], lymphocyte differentiation [17], and oncogenesis [18-21]. Emerging studies have shown that FBI-1 is strongly expressed in diffuse large B-cell lymphoma, follicular lymphoma, breast, lung, colon, prostate and bladder carcinomas [18,22]. However, its role in ovarian tumors has not been reported.

In the current study, we have illustrated a novel mechanism that may account for the aggressiveness and poor prognosis of human ovarian cancer. We showed that FBI-1 interacted and activated MT1-MMP, increased cell motility and invasion of ovarian cancer. FBI-1 is significantly up-regulated in advanced stages of ovarian cancer and associated with overall and disease free survival of patients with ovarian tumors.

## Results

### Overexpression of FBI-1 protein in ovarian tumors and cancer cell lines

**Figure 1:**
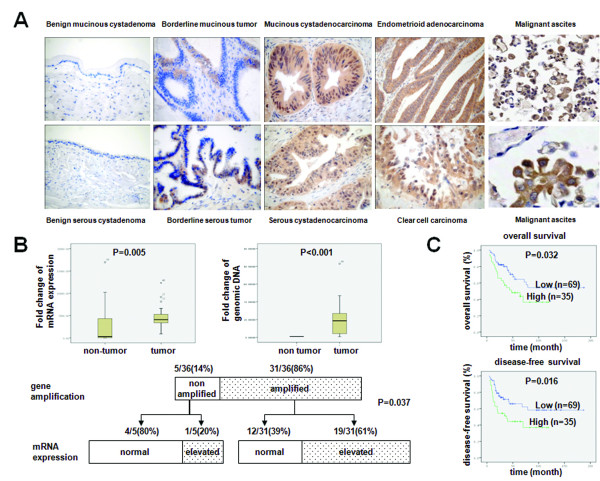
**FBI-1 was overexpressed in ovarian tumors and correlated with prognosis of patients**. *A*, Representative images of FBI-1 immunoreactivity in different ovarian tumor subtypes and malignant ascites (20×) (bottom right; 200×). *B*, mRNA expression (upper left panel) and gene amplification status (upper right panel) of FBI-1 in clinical samples and the correlation of gene amplification patterns with mRNA expression (lower panel). *C*, High expression of FBI-1 was associated with poor overall and disease-free survival.

The differential expression of FBI-1 in the various categories of ovarian tumors was shown in Figure 1A, and its association with clinical-pathological parameters was summarized in Table 1. All 10 benign cystadenomas (3 serous and 7 mucinous) were completely negative for FBI-1 staining, while 15 out of 19 (78.9%) borderline tumors (7 serous and 12 mucinous) showed weak and focal immunoreactivity (score ≤9). Among the 111 ovarian cancers, 82 (73.8%) cases displayed obvious overexpression of FBI-1 at a high level (score ≥12) both in the cytoplasm and nuclei. Significant higher expression of FBI-1 was found in borderline tumors (P < 0.001) and cancers (P < 0.001) when compared with benign counterparts. Expression of FBI-1 in cancers was also significantly higher than that in borderline tumors (P < 0.001). However, there was no significant difference among the four major histological types of ovarian cancers (Table 1).

**Table 1:** Association analysis between FBI-1 expression and the clinicopathological features of ovarian cancers

		Case assessed	Score (mean ± SE)	*p*-value
Diagnostic categories	Benign	10	0	0.000^a^
	Borderline	19	7.83 ± 0.92	0.000^a^, 0.000^b^
	Primary cancer	111	12.31 ± 0.37	0.000^a^
	Metastatic foci	63	13.47 ± 0.46	0.036^c^
	Malignant ascites	17	14.11 ± 0.87	0.021^c^
Histological types of ovarian cancer	Serous	43	12.53 ± 1.04	0.070^d^
	Endometrioid	36	11.82 ± 0.66	
	Clear cell	23	12.76 ± 0.53	
	Mucinous	9	12.11 ± 1.17	
Stage	Stage I	42	11.30 ± 0.67	0.012^e^
	Stage II-IV	69	13.38 ± 0.42	
Grade	Low (grade I)	32	12.22 ± 0.73	0.151^f^
	High (grade II and III)	79	12.39 ± 0.44	
Response to primary chemotherapy	Sensitive	78	12.14 ± 0.45	0.246^g^
	Resistant	18	13.22 ± 0.92	
Survival months	0.53~188.57 (average: 44.7)			

^a ^p Value reflects the comparison of each category vs. benign tumor.

^b ^p Value reflects the comparison of primary cancer vs. borderline.

^c ^p Value reflects the comparison of metastatic foci or malignant ascites versus primary cancer.

^d ^p Value reflects the comparison of clear cell carcinoma versus all the other histological types combined.

^e ^p Value reflects the comparison of stage I versus stage II-IV.

^f ^p Value reflects the comparison of low grade versus. high grade.

^g ^p Value reflects the comparison of chemosensitive versus chemoresistant.

**Figure 2:**
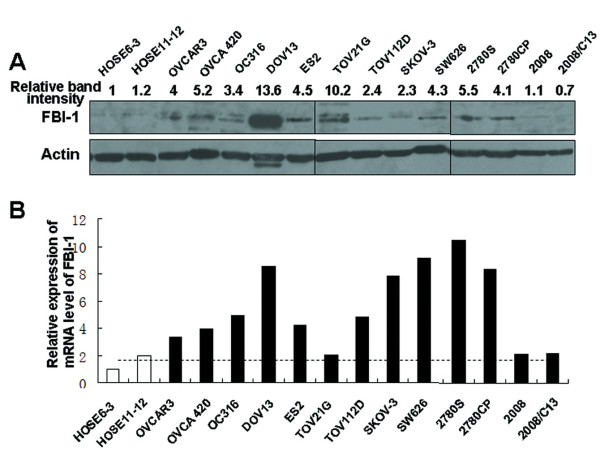
**FBI-1 expression was up-regulated in most of the ovarian cancer cell lines when compared with normal ovarian epithelial cell lines HOSE6-3 and HOSE11-12**. *A*, protein expression and *B*, mRNA level of FBI-1 as determined by western blot and qPCR respectively.

Western blot analysis showed that FBI-1 expression was up-regulated in 11 out of the 13 (84.6%) ovarian cancer cell lines (OVCAR3, OC316, DOV13, ES2, OVCA 420, SKOV-3, TOV21G, TOV112 D, SW626, 2780 S, and 2780CP) when compared with two nontumorigenic immortalized human ovarian surface epithelial cell (HOSE) lines (Figure 2A).

### Gene amplification contributes to the overexpression of FBI-1 mRNA

In a cohort of 36 pairs of clinical frozen samples, the mRNA level of FBI-1 was found to be significantly up-regulated in ovarian cancers when compared with corresponding non-tumor counterparts (P = 0.005) by qPCR (Figure 1B, left upper panel). Ten of the 13 (76.2%) ovarian cancer cell lines also displayed up-regulation of FBI-1 at mRNA level when compared with the average of HOSE6-3 and HOSE11-12 (Figure 2B), although a heterogeneous expression profile was observed.

To evaluate the mechanisms underlying the increase in FBI-1 expression, genomic DNA copy number of FBI-1 was further evaluated in these 36 pairs of patient samples using qPCR (Figure 1B, right upper panel). Remarkably, 31 out of 36 (86%) cancers displayed gene amplification of FBI-1 when compared with the corresponding non-tumor counterparts (Figure 1B, lower panel). Among the 31 amplified cases, 19 (61%) showed elevated mRNA levels. In the 5 non-amplified cases, increased mRNA expression of FBI-1 was only found in one case (20%). Spearman's rho test demonstrated that elevated RNA transcription of FBI-1 was closely correlated with its gene amplification (P = 0.037), suggesting that gene amplification was an important mechanism leading to the overexpression of FBI-1 in ovarian cancers.

### FBI-1 overexpression was significantly associated with aggressive tumor behavior and poor outcome

In addition to primary cancer samples, 63 metastatic foci in the lymph node, ligament, gut, and uterine serosa derived from advanced ovarian cancers and 17 ascitic samples were also used for evaluating FBI-1 protein expression by immunohistochemistry. Significant up-regulation of FBI-1 expression was detected in metastatic foci (P = 0.036) and malignant ascites (P = 0.021) (Figure 1A), compared with primary malignancies (Table 1). Moreover, higher FBI-1 expression was also found to be closely associated with advanced stage (stage II-IV) (P = 0.012) (Table 1), poor overall survival (P = 0.032) and disease-free survival (P = 0.016) (Figure 1C). Nevertheless, there was no significant correlation between FBI-1 immunoreactivity and histological grade (P = 0.151) or chemosensitivity (P = 0.246). Although increased FBI-1 significantly correlated with survival as mentioned above, multivariable analysis showed that expression of FBI-1 was not an independent predictor of overall survival (95% CI = 0.971-1.089, P = 0.344), or disease-free survival (95% CI = 0.896-1.080, P = 0.729). Together, our findings indicated that FBI-1 may affect the prognosis of patients with ovarian cancer via its effect on cancer progression.

### FBI-1 promoted cell migration and invasion of ovarian cancer with up-regulation of MT1-MMP

To further assess the function of FBI-1 on aggressive behavior of ovarian cancer cells, OVCA 420 (harboring wild-type *p53*) and SKOV-3 (possessing a single nucleotide deletion at point 267 at codon 90 of *p53*, which blocks p53 expression [23]) cells were transiently transfected with pEGFP-FBI-1, followed by performing cell migration and invasion assays. Among the various ovarian cancer cell lines, OVCA 420 and SKOV-3 showed average levels of FBI-1 expression.

**Figure 3:**
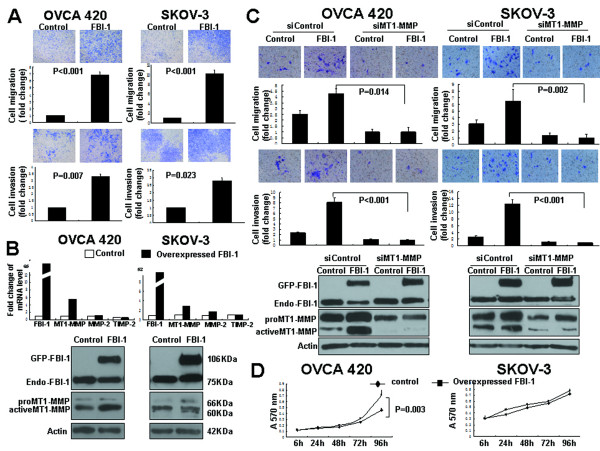
**Ectopic overexpression of FBI-1 promoted motility, invasiveness and proliferation of ovarian cancer cells**. *A*, *In vitro *migration (upper panel) and invasion assays (lower panel) in OVCA 420 (wild-type p53) and SKOV-3 (null p53) cell lines after transfection of pEGFP-FBI-1. *B*, mRNA expression of FBI-1, MT1-MMP, MMP-2 and TIMP-2 in cells after transfection of pEGFP-FBI-1 (upper panel). Western blot analysis of the expression of exogenous GFP-tagged FBI-1 (GFP-FBI-1) and endogenous FBI-1 (Endo-FBI-1) (as detected by anti-FBI-1 antibody) and MT1-MMP in cells after transfection of pEGFP-FBI-1 (lower panel). *C*, *In vitro *migration (upper panel) and invasion (middle panel) assays in OVCA 420 and SKOV-3 cells transiently transfected with pEGFP-FBI-1, or control vector combined with siRNAs of MT1-MMP or control. Western blot analysis (lower panel) of the expression of GFP-FBI-1 and Endo-FBI-1, and MT1-MMP in cells expressing FBI-1 transfected with siRNA of MT1-MMP. *D*, MTT assays revealed that overexpressed FBI-1 promoted cell growth of OVCA 420 which possessed wild-type *p53*, but not SKOV-3 cells with mutation and loss of expression of p53.

Up to 7 and 10-fold increase in cell migration were observed in OVCA 420 and SKOV-3 cells (both P < 0.001) (Figure 3A, upper panel) after transfection of pEGFP-FBI-1. About 3-fold increase in cell invasion was also observed in OVCA 420 (P = 0.007) and SKOV-3 (P = 0.023) cells (Figure 3A, lower panel). Next, the mRNA and protein expressions of FBI-1, MT1-MMP, MMP-2 and TIMP-2 were investigated. qPCR analysis showed that along with the induced FBI-1 mRNA expression, MT1-MMP, but not MMP-2 or TIMP-2, mRNA expression was increased by 3 to 6 fold after transfection of pEGFP-FBI-1 (Figure 3B, upper panel). Western blot analyses using anti-FBI-1 antibody also showed ectopic expression of exogenous GFP-tagged FBI-1 (106KDa) while no change in endogenous FBI-1 (75KDa) was found. An up-regulation of total and active form of MT1-MMP protein in both OVCA 420 and SKOV-3 cells after transfection of pEGFP-FBI-1 was also demonstrated (Figure 3B, lower panel). No significant change in MMP-2 and TIMP-2 expression was observed (data not shown).

To test whether MT1-MMP is involved in FBI-1-mediated cell migration and invasion, OVCA 420 and SKOV-3 cells were transiently transfected with pEGFP-FBI-1 and siRNA of MT1-MMP. Transient knockdown of MT1-MMP inhibited both basal and FBI-1-enhanced cell migration and invasion (Figure 3C; all P values < 0.05).

**Figure 4:**
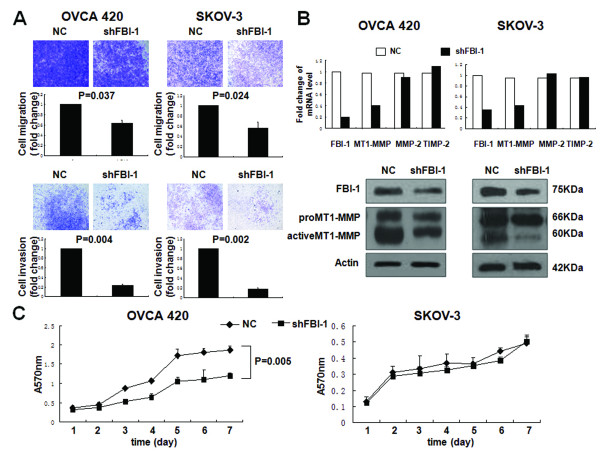
**Stable knockdown of FBI-1 with shRNA reduced motility, invasiveness and proliferation of ovarian cancer cells**. *A*, *In vitro *migration (upper panel) and invasion assays (lower panel) in OVCA 420 and SKOV-3 cell lines after stable knockdown of FBI-1. *B*, mRNA expression of FBI-1, MT1-MMP, MMP-2 and TIMP-2 in FBI-1 depleted cells (upper panel). Western blot analysis of the expression of FBI-1 (as detected by anti-FBI-1 antibody) and MT1-MMP in FBI-1 depleted cells (lower panel). *C*, Concurring with findings shown in Figure 3C, reduction of cell proliferation following FBI-1 knockdown was found exclusively in OVCA 420 cells with intact p53 function.

To further substantiate the notion that FBI-1 conferred to ovarian cancer cell motility and invasiveness, OVCA 420 and SKOV-3 cells with stably knockdown FBI-1 were established and subjected to transwell assays. Consistent with previous results, for both cell lines, a reduction in the number of migrated and invaded cells (p < 0.05) was significantly associated with a decrease in MT1-MMP mRNA and protein levels, in particular the active form of the MT1-MMP (Figure 4A, B).

### Knockdown of FBI-1 reduced ovarian cancer cell mobility irrespective of p53 status

**Figure 5:**
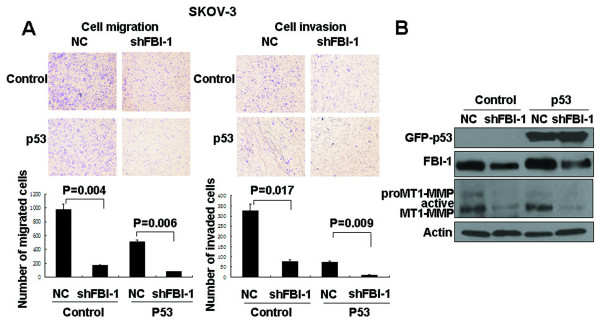
**Introduction of wild type p53 into SKOV-3 cell lines with stable FBI-1 knockdown (shFBI-1) and negative counterpart (NC) led to *A*, significant reduction in cell migration and invasion in association with *B*, reduced expression of FBI-1 (as detected by anti-FBI-1 antibody) and MT1-MMP**. Bars, means of migrated or invaded cell numbers ± SD obtained from independent experiments.

As OVCA 420 and SKOV-3 have different *p53 *status, it is likely that FBI-1 may affect the cell migration and invasion in a p53-independent manner. To test this hypothesis, migration and invasion assays were performed on SKOV-3 cells harboring stable knockdown FBI-1 with or without re-induction of wild-type p53. In both stable shFBI-1 cells with or without re-expression of wild-type p53, similar decreases in cell invasion and migration (p < 0.05) were observed, which were again accompanied by a reduction of MT1-MMP expression compared with the negative control (NC) (Figure 5A, B). Together, these results suggested that FBI-1 play an important role in ovarian cancer cell invasion, migration, and metastasis, and this function of FBI-1 is mediated at least in part through its regulation of MT1-MMP expression but is independent of *p53 *status.

### FBI-1 directly interacts with the promoter of MT1-MMP and enhanced its expression

**Figure 6:**
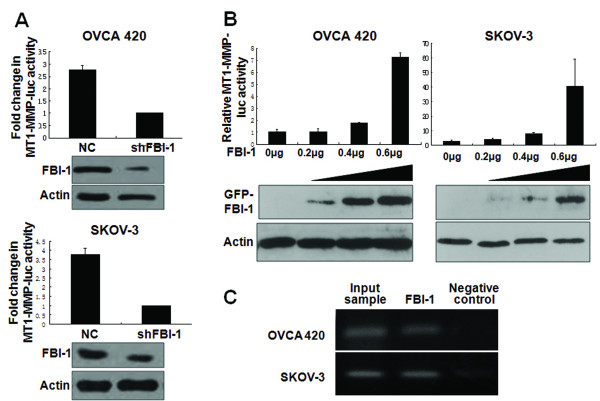
**Dual luciferase assays demonstrated down-regulation of MT1-MMP transcription in *A*, OVCA 420 and SKOV-3 with stable shFBI-1 knockdown**. Up-regulation of MT1-MMP transcription was found in *B*, OVCA 420 and SKOV-3 cells after transfection of pEGFP-FBI-1 in a dose-dependent manner (upper panel); exogenous GFP-tagged FBI-1 (GFP-FBI-1) expression was determined by Western blot analysis using anti-GFP antibody (lower panel). *C*, ChIP assays in OVCA 420 and SKOV-3 cell lines. Protein-DNA complexes are immunoprecipitated either with anti-FBI-1 antibody or negative control, followed by PCR with primers specific to MT1-MMP promoter sequence and agarose-gel electrophoresis for visualization. Total lysates were used as the input samples and positive control.

The ability of FBI-1 to modulate both MT1-MMP mRNA and protein levels suggested that FBI-1 may regulate MT1-MMP expression at transcriptional level. FBI-1 binding sites have recently been characterized [18], and three putative FBI-1 consensus sequences could be identified within ~450bp upstream of the transcription start site of the *MT1-MMP *gene [24]. A DNA fragment corresponding to an approximately 600bp of the 5'-flanking sequence of *MT1-MMP *was subcloned upstream of a luciferase reporter assay and used for gene promoter analysis. Firstly, we investigated whether FBI-1 would affect *MT1-MMP *gene transcription activity in OVCA 420 and SKOV-3 cells after stable FBI-1 knockdown. FBI-1 depletion reduced the *MT1-MMP *promoter activity in both cell lines by 3 and 4-fold when compared with the negative controls, respectively (Figure 6A). Secondly, the pGL3-Basic-*MT1-MMP*-Luc reporter was co-transfected with increasing amounts of pEGFP-C3-FBI-1 construct. The result showed that FBI-1 efficiently enhanced *MT1-MMP *promoter activity in a dose-dependent manner (Figure 6B). Yet, FBI-1 expression showed no significant effect on *MMP-2 *promoter by luciferase reporter assay (data not shown).

In an attempt to further elucidate the induction of *MT1-MMP *transcription by FBI-1, a luciferase reporter construct containing 94bp of the human *MT1-MMP *promoter (pGL3-Basic-shortMT-MMP-luc) was applied for gene promoter analysis. Theoretically, there is no FBI-1 DNA binding site in this short MT-MMP-luc sequence [18,24]. Our result showed that overexpression of FBI-1 could not enhance the transcriptional activity of this short form of *MT1-MMP *promoter (data not shown), further supporting the specificity of the transcription regulatory effect of FBI-1 on *MT1-MMP *gene.

We next examined whether FBI-1 can bind to the endogenous *MT1-MMP *promoter *in vivo *using the ChIP assays. Total lysates from parental OVCA 420 and SKOV-3 cells were used as the input samples and positive control, while immunoprecipitated lysates without antibody served as the negative controls. As shown in Figure 6C, FBI-1 bound directly to the promoter region of *MT1-MMP *gene.

### FBI-1 promoted ovarian cancer cell proliferation in a p53-dependent manner

In addition, we also investigated whether FBI-1 could affect cell proliferation using MTT assay. Introduction of FBI-1 into OVCA 420 cells with wild-type *p53 *resulted in an approximately 2-fold enhancement in cell proliferation (P = 0.003) (Figure 3D). In contrast, there was no significant effect in SKOV-3 cells which have no p53 expression. Consistent with these findings, stable silencing of FBI-1 in OVCA 420 cells led to a significant decrease in cell proliferation, whereas no significant changes were observed in SKOV-3 cells (Figure 4C). The results indicated that the FBI-1 may modulate ovarian cancer cell proliferation in a p53-dependent manner.

## Discussion

Since ovarian cancer is often asymptomatic until it has extensive extra-ovarian spread, the high lethality of this disease is at least partly attributed to the fact that patients are often at an advanced stage at the time of first diagnosis [25]. Therefore, a more precise understanding of the genetic mechanisms that control cancer cell dissemination is important. In this report, we established for the first time that vast majority of ovarian cancers produced aberrantly high levels of FBI-1 when compared with benign or borderline tumors. Overexpression of FBI-1 was also observed in most of the ovarian cancer cell lines. Clinicopathological analysis indicated that high expression of FBI-1 was associated with advanced stage, shorter overall and disease-free survival. The cancer cells at metastatic foci and malignant ascites stemmed from advanced diseases expressed significantly higher levels of FBI-1 when compared with the primary cancers, supporting a role of FBI-1 in the dissemination of ovarian cancer. Using cell line models, we further provided evidence supporting that FBI-1 plays a significant role in migration, invasion, and proliferation of ovarian cancer.

FBI-1 is encoded by the *ZBTB7A *gene at chromosome 19p13.3. To date, little is known about the mechanism leading to *FBI-1 *up-regulation in cancer. In non -Hodgkin B-cell lymphoma, t(14;19)(q32;p13.3) translocation was reported and aberrant activation of upstream regulators was speculated to be involved in its up-regulation[26]. Recently, *FBI-1 *gene amplification was found to be a frequent event in non-small cell lung cancer [22]. Indeed, a remarkable proportion of our ovarian cancers (86%) displayed more than two-fold amplification of *ZBTBZA *gene and the gene amplification status was significantly correlated with its elevated expression at transcriptional level.

Previous reports suggested that the key role of FBI-1 in oncogenesis is to act as a potent inhibitory regulator of the tumor suppressor gene *p14^ARF^*, subsequently leading to MDM2 constitutive activation and p53 degradation [18,27]. Taking advantage of ovarian cancer cell lines with different *p53 *status, we proved that FBI-1 could enhance cancer cell invasion and migration in a p53-independent manner. This hypothesis was confirmed by wild-type p53 manipulation study. It is likely that FBI-1 has more target genes than originally anticipated. Indeed, studies have suggested that FBI-1 could repress retinoblastoma gene, another well-known tumor suppressor gene [20] and could activate fatty-acid synthase gene (FASN) [28].

Unlike other epithelial carcinomas, which mainly metastasize through vasculature, "shedding" and "seeding" of ovarian cancer cells from the primary tumor into peritoneal cavity are also common [29]. These tumor dissemination processes involve detachment of tumor cells and degradation of the basement membrane. Thus, proteolytic activity becomes extremely important in ovarian cancer metastasis because it facilitates the breakthrough and invasion of the mesothelial monolayer and collagen-rich extracellular matrix as well. MT1-MMP/MMP-2/TIMP-2 axis is considered as a central determinant in these processes. Even though the posttranscriptional regulations of MT1-MMP have been extensively studied [30,31], knowledge about its transcriptional regulations remain limited. The GC-rich promoter of MT1-MMP is different from the other MMPs which are composed of a conserved TATA box at promoter region [24]. Recently, transcription factors Sp1, Sp3 and Egr-1 have been identified as potent regulators of MT1-MMP expression in prostate cancer, endothelial and glomerular mesangial cells [24,32,33]. Our present findings support that FBI-1 can directly bind to the promoter of *MT1-MMP*, but not *MMP-2*, and induce MT1-MMP expression in both transcription and protein levels, in a dose dependent manner leading to cell migration and invasion. Induction of MT1-MMP transcription by FBI-1 by mutating the potential binding sites in the MT1-MMP promoter will be performed in our future study. On the other hand, the findings of MTT assays on the two ovarian cancer cell lines harboring different *p53 *status suggested that the effect of FBI-1 on cell proliferation might be restricted in cells with intact p53 function, consistent with findings from a previous report [34].

In conclusion, our data showed that FBI-1 promotes aggressive cancer cell phenotype and its overexpression is associated with poor clinical outcome. The role of FBI-1 in ovarian cancer development and progression also makes it a potential target for therapeutic intervention in ovarian cancer treatment.

## Methods

### Patient samples and cell lines

220 archival clinical samples (Table 1), including paraffin-embedded tissues of 10 ovarian benign cystadenomas (ages, 20~77 years; mean age, 32 years), 19 ovarian borderline cystadenomas (ages, 20~46 years; mean age, 30 years), 111 ovarian cancers (ages, 23~78 years; average age, 50 years) and corresponding metastatic foci in 63 cases, as well as 17 malignant ascites obtained from stage III/IV ovarian cancer patients, were collected at the time of surgical resection from 1987 to 2005 at Department of Pathology, Queen Mary Hospital, the University of Hong Kong. In addition, 36 paired samples of ovarian cancers and their corresponding normal fallopian tubes and/or contralateral ovaries were processed for snap-frozen blocks followed by storage at -80°C and total RNA and genomic DNA extractions. Both collection and the use of such patient samples were approved under the Institutional Ethics Review Board. All diagnoses have been confirmed by two pathologists using criteria of the International Federation of Gynecology Oncology (FIGO).

Culture conditions of two immortalized normal human ovarian surface epithelium cell lines (HOSE6-3 and HOSE11-12) and 13 ovarian cancer cell lines (OVCA420, OVCAR-3, SKOV-3, 2780 S, 2780CP, 2008, 2008/C13, SW626, ES2, OC316, DOV13, TOV21G and TOV112D) were described previously [35-38]. Cell lines used in the present study were in culture for less than 6 months.

### Immunohistochemistry and western blot

Immunohistochemical staining for FBI-1 (1:400; Abcam Inc, Cambridge, UK; ab36606) was carried out on 220 paraffin-embedded clinical samples with EnVision_Dual Link System (K4061; Dako, North America, Carpinteria, CA, USA) [36]. For negative control, primary antibody was replaced with phosphate buffer saline (PBS). Sections were assessed for both staining intensity and percentage, and the final score was determined as described [35].

For western blot, antibodies specific to FBI-1 (1:600; Abcam), MT1-MMP (1:1000; Sigma; MS221106), p53 (1:1000; Dako; clone DO-7), and GFP (1:1500; Santa Cruz; sc-9996) were used to detect the immunoreactivity according to standard procedures [36]. Our plasmid expressing FBI-1 was tagged with GFP. GFP is a protein composed of 238 amino acids (around 31kDa). Endogenous FBI-1 (75kDa) and GFP-tagged FBI-1 (106kDa) could therefore be differentially detected in 7.5% SDS-PAGE gels using anti-FBI-1 antibody.

### RNA and DNA preparation, quantitative real-time PCR (qPCR)

According to the manufacturer's instruction, total RNA and genomic DNA were isolated from frozen tissues and cell lines by using TRIzol reagent (Invitrogen, San Diego, CA, USA) or phenol/chloroform (Invitrogen) respectively. First-strand cDNA was synthesized with oligo-dT primer and SuperScript III Reverse Transcriptase kit (Invitrogen). ABI Prism 7700 Sequence Detection System and SYBR-green PCR master mix (Applied Biosystems) were used for qPCR as described [35].

The primers used for mRNA level evaluation are as follows: FBI-1, forward, 5'-TCTGCGAGAAGGTCATCC-3', and reverse, 5'-CGTAGTTGTGGGCAAAGG-3'[22]; MT-MMP, forward, 5'-ACGGAGGTGATCATCATTGAGG-3' and reverse, 5'-AGATGGGGCTGGACAGACACA-3'[37,39]; MMP-2, forward, 5'-GGCCCTGTCACTCCTGAGAT-3' and reverse, 5'-GGCATCCAGGTTATGGGGGA-3'[36,40]; TIMP-2, forward, 5'-GCGGTCAGTGAGAAGGAAGTGG-3', and reverse, 5'-CTTGCACTCGCAGCCCATCTG-3'[41]; GAPDH, forward, 5'-TCCATGACAACTTTGGTATCGTG-3' and reverse, 5'-ACAGTCTTCTGGGTGGCAGTG-3'[35]. Each sample was run in duplicate and normalized with GAPDH.

FBI-1 gene amplification was also assessed by qPCR using the following primers: forward, 5'-GAACGAGGGTTTAGTGCA-3' and reverse, 5'-CGAGCTGTTCTGGAGAGA-3'[22]. TRAT1, a single copy gene in ovarian cancer [42], (forward, 5'-CATGTCAGGTAAGTGGCATT-3'; reverse, 5'-GGGTCTTCTCGTTAGGACTTAG-3'), was served as an internal control. Each sample was verified in duplicate. Scoring ≥ two-fold difference in ovarian cancer samples compared with the corresponding normal counterpart indicated gene amplification.

### Plasmids

The fell-length cDNA of human FBI-1 was amplified from OVCAR3 cells by PCR using FastStart Taq DNA polymerase (Roche Applied Science, Indianapolis, USA) and the primers were forward, 5'- CCCAAGCTTGGGATGGCCGGCGGCGTGGACGGC-3' and reverse, 5'- GCCTTAAGGCTTAGGCGAGTCCGGCTGTGAA-3'. PCR product was cloned into pEGFP-C3 vector with Hind III and EcoR I. The integrity and accuracy of insert was confirmed by sequencing.

pEGFP-C1-p53 construct expressing wild-type p53 and empty vehicle were generous gifts from Dr. Wilson Ching (Department of Anatomy, The University of Hong Kong, HKSAR, China). Promoter-luciferase reporter genes, pGL3-Basic-MT1-MMP-Luc (bp -500/+112) and pGL3-Basic-shortMT-MMP-luc (bp -180/+112) were kindly provided by Professor Constance E. Brinckerhoff [24,43] (Department of Biochemistry and Medicine, Norris Cotton Cancer Center, Dartmouth Medical School, Lebanon, NH 03756, USA). pGL2-Basic-MMP-2-Luc (full length of promoter of human MMP-2) was a generous gift from Dr. Etty N. Benveniste (Department of Cell Biology, University of Alabama at Birmingham, Birmingham, Alabama).

### Transient transfection and luciferase reporter assay

For ectopic expression of FBI-1 and p53, or knockdown of MT1-MMP, cells (OCVA 420 and SKOV-3) were seeded at 90% confluence one day before transient transfection with various amounts of pEGFP-FBI-1, pEGFP-p53, siMT1-MMP (Ambion, USA), or controls using Lipofectamine™2000 (Invitrogen) according to manufacturer's instruction. Twenty-four hours after transfection, cells were collected followed by migration and invasion assays and proliferation analysis. To assess the transcriptional activities of MT1-MMP and MMP-2, 1 × 10^5 ^cells were grown in 24-well plates and transiently co-transfected with indicated dosage of pEGFP-FBI-1 construct and pGL3-Basic-MT1-MMP-Luc or pGL2-Basic-MMP-2-Luc reporter plasmids. pRL-SV40-Luc was used as internal control, whereas corresponding empty vectors were served as negative controls. After 48 h incubation, cells were lysed for luciferase activity analysis using the Dual-Luciferase Reporter Assay System (Promega, Madison, WI). *Renilla *luciferase activities were used to normalize the transfection efficiency. Each experiment was repeated twice in duplicate wells.

### Establishment of FBI-1 stable knockdown cells

For stable silencing, human FBI-1-specific shRNA (SureSilencing shRNA constructs) and control shRNA vector (pGeneClip™ puromycin vector) were purchased from SuperArray (SABioscicences Corporation, Frederick, USA), transfected into OVCA 420 and SKOV-3 cells, and selected with puromycin as published previously[36]. Knockdown efficiency was confirmed by qPCR and immunoblotting analysis.

### In Vitro migration and invasion assays

To determine the impact of FBI-1 on cell mobility, transient overexpressing or stable knockdown FBI-1 cells and their control cells were counted and equally plated on the upper compartments of the 24-wells, 8-μm pore size Transwell chambers (BD Biosciences, San Jose, CA). Cell migration and invasion assays were performed with self-coated Gelatin and Matrigel, respectively. After 24 h, the migrated and invaded cells on the lower surface of membrane were fixed and stained with methanol mixed crystal violet. All assays were run in independently three times.

### Proliferation assay

Effect of FBI-1 with or without wild type p53 on cell proliferation was determined by 3-[4,5-dimethylthiazol-2-yl]-2,5-diphenyl-tetrazolium bromide (MTT) assay as previously described [36]. Every experiment was repeated twice in triplicate wells, separately.

### Chromatin immunoprecipitation (ChIP)

Confluent OVCA 420 and SKOV-3 cells grown on 10-cm dish were cross-linked by adding 1% formaldehyde for 10 min. After stopping the reaction with 1.25 M glycine solution and washing with PBS, cells were suspended in RIPA buffer (150 mM NaCl, 1% NP-40, 0.5% sodium deoxycholate, 0.1% SDS, 50 mM Tris, and 5 mM EDTA) containing protease inhibitor cocktail and sonicated until most of the DNA fragments ranged from 500 to 1000bp. 50 μl of soluble lysate was saved as 'input sample'. The rest of the lysate was pre-cleared with protein A/G beads and salmon sperm DNA. Anti-FBI-1 antibody and 30 μl protein A/G beads were then added to the pre-cleared samples. Samples with no antibody incubation were used as negative control. Beads were centrifuged and washed with RIPA buffer, IP buffer (0.5 M LiCl, 0.1 M Tris, 1%NP-40, and 1% sodium deoxycholate), and then RIPA buffer. The bound material was eluted from the beads at 85°C for 10 min in elution buffer (0.1 M NaHCO_3 _and 1%SDS) and the RNA and protein was digested using RNase A and proteinase K, respectively. DNA was precipitated and purified by using phenol/chloroform. The following primers were used to detect MT1-MMP promoter sequences, which is designed using Primer3 (v.0.4.0) software based on the sequence of luciferase reporter construct [24]: forward, 5'-CCGACAGCGGTCTAGGAAT-3', reverse, 5'- AGACAACGGGAGGGTCTTG-3'.

### Statistical analysis

The clinical-pathological features and the data from *in vitro *assays were analyzed using Statistical Package for Social Science 15.0 for windows (SPSS Inc., Chicago, IL, USA) software. Spearman's rho test, Kruskal-Wallis rank test, Mann-Whitney test, Kaplan-Meier method (using log-rank test), Cox's regression model were used to assess correlation, difference of multiple groups, difference between two groups of non-parametric data, survival probability and multivariate survival analysis, respectively. P value <0.05 was considered to be significant.

## Competing interests

The authors declare that they have no competing interests.

## Authors' contributions

LLJ designed and performed the experiments, interpreted the results and prepared the manuscript under the supervision of ANYC, MKYS and OGWW who also conceived the study and critically revised the manuscript. ANYC, KFT and HYSN provided the clinical material while ANYC also facilitated laboratory settings for the experiments. WFL and XFL provided cell lines and contributed to the manuscript editing. ESYW and HYC provided the technical assistance for experiments. All authors approve the final version of the manuscript.
